# Intratumoral heterogeneity of cancer driver genomic alterations in myxoid liposarcomas

**DOI:** 10.1002/cncr.35937

**Published:** 2025-06-09

**Authors:** Adrian Schmid, Anja E. Eisenhardt, Balazs Bogner, Alexander Runkel, Ute Lausch, Thomas Pauli, Laura N. Antolini, Anika Boneberg, Jurij Kiefer, Peter Bronsert, Melanie Boerries, Steffen U. Eisenhardt, David Braig

**Affiliations:** ^1^ Department of Plastic and Hand Surgery Medical Center ‐ University of Freiburg Freiburg im Breisgau Germany; ^2^ Department of Radiology Medical Center ‐ University of Freiburg Freiburg im Breisgau Germany; ^3^ Institute of Medical Bioinformatics and Systems Medicine Medical Center ‐ University of Freiburg Freiburg im Breisgau Germany; ^4^ Institute for Surgical Pathology Medical Center ‐ University of Freiburg Freiburg im Breisgau Germany; ^5^ Tumorbank Comprehensive Cancer Center Freiburg Medical Center ‐ University of Freiburg Freiburg im Breisgau Germany

**Keywords:** clonal evolution, myxoid liposarcoma, next‐generation sequencing, soft tissue sarcoma, tumor heterogeneity

## Abstract

**Background:**

Myxoid liposarcomas (MLS) are rare malignant mesenchymal tumors characterized by specific translocations t(12;16) and t(12;22) with limited additional driver mutations, most notably in *PIK3CA* and the *TERT* promoter. *PIK3CA* is considered a promising therapeutic target. However, effective treatments require the uniform presence of mutation throughout the tumor. Therefore, this study evaluated intratumoral heterogeneity of driver mutations in MLS.

**Methods:**

In total, 170 samples from 20 tumors (12 patients) were analyzed using an MLS‐specific next‐generation sequencing (NGS) panel. This included detecting the t(12;16) and t(12;22) translocations and known driver mutations.

**Results:**

Patient‐specific t(12;16) or t(12;22) translocations were detected in all 20 tumors (159 of 170 samples; 94%) and remained identical in primary tumors, recurrences, and metastases. *TERT* promoter mutations were identified in 17 of 20 tumors (85%) and were distributed similarly across samples. In contrast, *PIK3CA* mutations were present in only 66 of 170 samples (39%), with these and the remaining driver mutations localized only in subclones within individual tumors.

**Conclusions:**

Therapies that target *PIK3CA* are unlikely to succeed because of its limited subclonal distribution. In contrast, the ubiquitous presence of t(12;16), t(12;22), and *TERT* promoter mutations across MLS tumors suggests that these are more effective therapeutic targets for future treatment strategies.

## Abstract 2

Myxoid liposarcomas (MLS) are rare, malignant, mesenchymal tumors characterized by specific translocations t(12;16) and t(12;22) with limited additional driver mutations, most notably in *PIK3CA* and the *TERT* promoter. This study demonstrated that the previously favored optional target *PIK3CA* appears to be much less suitable as a therapeutic option because of intratumoral heterogeneity and that future research should focus on the translocations or the *TERT* promoter.

## INTRODUCTION

High‐grade soft tissue sarcomas (STS) are rare malignant tumors that arise from mesenchymal tissue and are associated with a high mortality rate. The most common STS include liposarcomas, leiomyosarcomas, and pleomorphic sarcomas and are typically found in the extremities and trunk.^1^ For localized STS, surgical resection combined with a multimodal treatment approach can be curative. However, approximately one third of patients with STS will develop distant metastases.^2^

At the molecular level, STS are divided into two categories. The first category includes sarcomas with complex karyotypes and nonspecific genetic alterations, such as leiomyosarcomas or myxofibrosarcomas. The second category includes sarcomas with characteristic translocations driving the disease.^3^ For example, myxoid liposarcomas (MLS) harbor one of two specific translocations associated with a few additional hotspot mutations.^4^ In greater than 90%–95% of patients with MLS, the reciprocal translocation t(12;16)(q13;p11) results in a fusion of the *DDIT3* and *FUS* genes. Less commonly, the translocation t(12;22)(q13;q11–12) fuses *DDIT3* with *EWSR1*. These patient‐specific translocations occur within defined regions and initiate tumor growth.^5^, ^6^

STS exhibit significant intratumoral heterogeneity.^7^ The extent to which this heterogeneity is reflected at the genomic level remains unclear. Tumor heterogeneity can cause misinterpretations, especially regarding treatment options and response. Although no targeted therapy for locally advanced or metastatic MLS has produced promising results, there are several potential targets for which targeted therapy already exists.^8^ For example, the PI3K inhibitor Alpelisib can be used to treat certain breast cancer types.^9^ Because *PIK3CA* variants have been described in approximately 30% of patients with MLS, a potential treatment benefit could be derived from this. However, because of intratumoral heterogeneity, the overall prevalence of individual mutations can be highly variable; therefore, only a limited response to targeted therapy would be expected.^6^ In this context, a better understanding of the mutational landscape within MLS could lead to direct therapeutic benefits.

We investigated the heterogeneity of driver mutations in tumor samples from patients with histopathologically confirmed MLS. By analyzing the heterogeneity of recurrences and metastases, we were able to infer the clonal evolution of MLS. The results may help to identify targeted therapies with a high chance of therapeutic success.

## MATERIALS AND METHODS

### Study population

In total, 170 tumor samples from 20 individual tumors were obtained from 12 patients with MLS who were treated at the Comprehensive Cancer Center Freiburg (Table 1) and analyzed for genetic heterogeneity.

**TABLE 1:** Study population.

	No. of patients or tumors	%
Total no. of patients	12	100.0
Total no. of tumors	20	100.0
Sex (patients)		
Men	11	92.0
Women	1	8.0
Age at first diagnosis: Mean ± SD, years	49.3 ± 12.5	
Metastasis status (patients)		
M0	6	50.0
M1	6	50.0
Grade (tumors)		
1	5	25.0
2	12	60.0
3	3	15.0
Radiotherapy (tumors)		
Neoadjuvant	8	40.0
Adjuvant	10	50.0
Chemotherapy (tumors)		
Neoadjuvant	0	0.0
Adjuvant	2	10.0
Anatomic distribution (tumors)		
Upper extremity	1	5.0
Lower extremity	14	70.0
Trunk	5	25.0
Head and neck	0	0.0

*Note*: The table includes the study population with myxoid liposarcoma (*n* = 12). Grade, radiation, chemotherapy, and anatomic distribution are listed for each tumor/metastasis status.

Abbreviation: SD, standard deviation.

### Ethics, consent, and permission

The study followed the Declaration of Helsinki and was approved by the Ethics Committee of the Albert‐Ludwigs‐University Freiburg (study numbers 236/16 and 21‐1735). All participants provided informed consent for analysis and anonymized data publication (registered as trial DRKS‐ID DRKS00009587).

### Tissue sampling

Tumor tissue from patients with MLS was collected postresection, formalin‐fixed, and stored at the Institute of Surgical Pathology, University of Freiburg. The tissue was sliced and stained with hematoxylin and eosin. The number of slices depended on the tumor size. One representative tissue sample from each slice was used for DNA extraction.

### DNA isolation

#### FFPE tissue

DNA was extracted using the QIAamp DNA FFPE (formalin‐fixed, paraffin embedded) Tissue Kit (Qiagen GmbH) following the manufacturer's protocol. Eight 10‐µm sections (approximately 25 mg) were digested with proteinase K at 56°C for 72 hours before proceeding with the protocol. The DNA was eluted in 80 µl nuclease‐free water.

#### Whole blood and leukocytes

DNA was isolated using the DNeasy blood and tissue kit (Qiagen GmbH) according to the *purification of total DNA from animal blood or cells (spin‐column) protocol* starting with 100 µl of whole blood. Subsequent steps were performed according to the manufacturer's instructions. The DNA was eluted in 200 µl nuclease‐free water.

### Library preparation, target enrichment, and sequencing

Libraries were prepared using the NEBNext Ultra II FS DNA Library Prep Kit for Illumina (New England Biolabs). Sixty nanograms of input DNA was enzymatically fragmented to approximately 150 base pairs (bp) by incubating for 25 minutes at 37°C, followed by eight cycles of polymerase chain reaction (PCR).

Target enrichment was conducted using IDT xGen Panels and a previously published, *MLS‐specific* panel (approximately 37 kilobase pairs).^6^ Enrichment was performed according to the xGen Hybridization and Wash Kit protocol (Integrated DNA Technologies) using double capture with pooled libraries (from 500 ng to 1 µg per pool). For postcapture PCR, 16 cycles (first lockdown) and nine cycles (second lockdown) were used.

Purified library fragment lengths were measured using the Tape Station Agilent D500 (Agilent Technologies), and concentrations were determined using quantitative PCR on the LightCycler 480 System (Roche) with the NEBNext Library Quant Kit (New England Biolabs). Sequencing was conducted on an MiSeq system (Illumina Inc.) with paired‐end reads (MiSeq V2, 300‐cycles).

### Quantification of DNA and fragment length

DNA concentrations were measured after DNA isolation, library preparation, and hybridization capture using the Invitrogen Qubit 3 Fluorometer (Invitrogen) with the dsDNA HS (double‐stranded DNA, high‐sensitivity) Assay Kit (0.2–100 ng).

### Bioinformatical pipelines and analysis

To analyze structural variants (SVs) and identify novel translocations, samples were processed using a custom script. Reads were aligned to the human genome (hg38) using the Burrows–Wheeler Aligner (BWA)‐MEM (version 0.7.17‐r1188).^10^ SAMtools (version 1.16.1) was used for conversion to binary (BAM) format, sorting, duplicate marking, and indexing.^11^ SVs were detected using FACTERA (Fusion and Chromosomal Translocation Enumeration and Recovery Algorithm; version 1.4.4).^12^ Therefore, the provided hg19 target file was converted to hg38 using LiftOver (UCSC Genome Browser Database; University of California, Santa Cruz).^13^ To verify newly identified translocations and to check other samples from the same patient for these SVs, the original FASTQ files were further processed. An individual fusion sequence was designed for each potential breakpoint, reaching 180 bp upstream and downstream, depending on orientation. These sequences were combined into patient‐specific fusion FASTA files. Raw FASTQ files were mapped again to these fusion FASTA files with BWA‐MEM (version 0.7.17‐r1188)^10^ and processed using SAMtools (version 1.16.1),^11^ as described above. The resulting BAM files were checked for positive alignment to the individual fusion sequences. Reads were considered positive if they had an alignment of at least 15 bp upstream and downstream of the breakpoint, with a maximum of one mismatched base within this 30‐bp sequence.

For single nucleotide variant (SNV) analysis, FASTQ files were uploaded to Illumina BaseSpace and processed using the DRAGEN Somatic App (version 4.2.7; both from Illumina Inc.) with matched normal analysis. Variants required a minimum variant allele frequency of 2.5%, sequencing depth ≥50 x, and no more than one false‐positive read in matched normal DNA. For heterogeneity analysis, each tumor segment's BAM file was analyzed separately. BAM files were manually reviewed for known variants using the Integrative Genomics Viewer (version 2.17.4; Broad Institute).^14^ Only variants that were present in at least one tumor segment with a minimum variant allele frequency of 2.5% were included in the analysis. Sequencing metrics were calculated using CollectHsMetrics in Picard Tools (version 2.27.5; Broad Institute).15

### Statistical analysis and figures

Statistical analyses and graph generation were performed using GraphPad Prism (version 10.2.3; GraphPad Software). Chord diagrams were generated with R Statistical Software (version 4.1.2; R Core Team)^16^ using RStudio (version 2023.06.1, Build 524; R Studio Team),^17^ and the R package circlize (version 0.4.16; R Core Team).^18^

### Analysis of clinical imaging data

Primary tumors and their respective metastases from three patients were manually segmented using the anatomy visualizer tool in syngo.via (Siemens Healthineers). The tumors from each patient were integrated into a whole‐body positron emission tomography‐computed tomography mask, which was previously acquired during routine clinical diagnostics. To enable a comprehensive visualization of disease progression and metastatic spread over time, the different tumors at various time points were highlighted with distinct colors.

## RESULTS

### Mutational profiling and genomic heterogeneity in MLS tumors

One hundred seventy samples of 20 individual tumors from 12 patients with MLS who were treated at the Comprehensive Cancer Center Freiburg were examined regarding their mutational landscape and intratumoral heterogeneity in cancer genomic drivers.

Tumor tissue from patients with MLS patients were processed as described above. Leukocyte DNA was available from each patient as a matched normal control sample. The subtype‐specific *MLS panel* was used for target enrichment.^6^ On‐target rates were consistently high with no significant differences between samples (see Figure S1A). The mean coverage varied between individual tumors, ranging from 40x to nearly 1000x. The coverages of matched normal samples were significantly higher (coverages from 550x up to 1161x; paired *t*‐test) than coverages of corresponding tumor samples sequenced in the same sequencing run.

The tumors were segmented for heterogeneity analysis (from one to 20 individual samples per tumor), and each sample was analyzed separately. For three patients, additional tissue from metastases was available.

**FIGURE 1:**
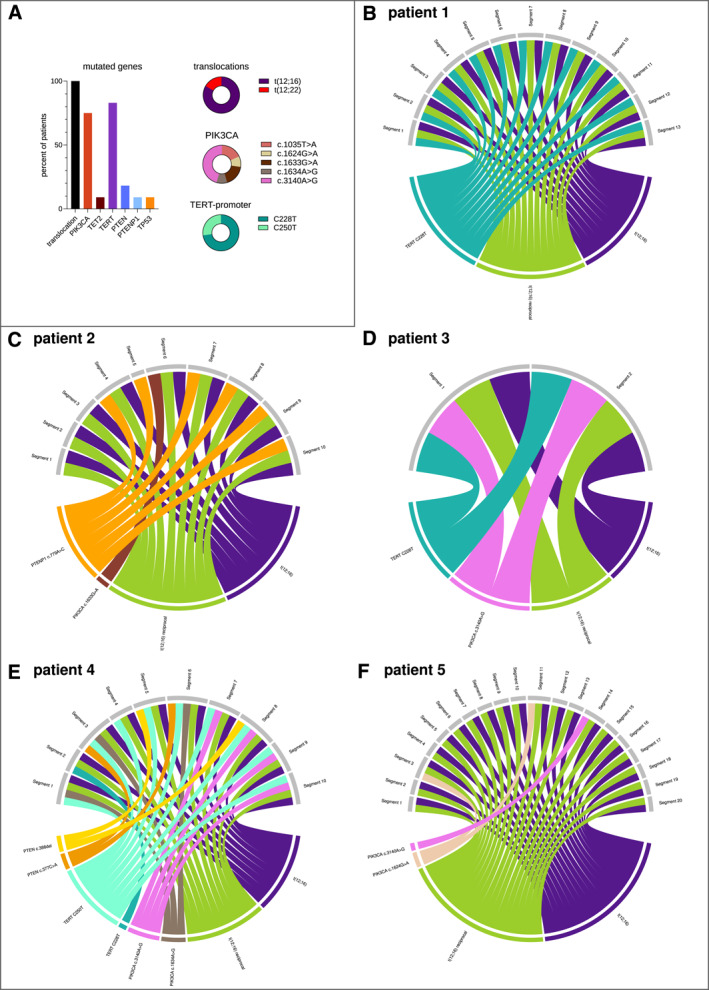
Mutational profiling and tumor heterogeneity in MLS, part 1. (A) The detected mutations after subtype‐specific target enrichment in MLS tumors are illustrated. Every pathologically confirmed MLS harbored an entity‐specific fusion event. Mutations in the *TERT* promoter region occurred in approximately 85% of tumors, with the C228T variant accounting for the largest proportion. Variants in *PIK3CA* were observed in 70% of tumors. (B–F) The figures illustrate intratumoral heterogeneity in t(12;16)‐positive MLS tumors, with only one tumor analyzed per patient. The detected variants for each tumor are depicted in a chord diagram. Each gray bar represents a segment, whereas the variants are indicated as colored bars. The colors are kept equal for all tumors. The colored links indicate which variant was detected in which sample. (B) The resected tumor from patient 1 was cut into 13 segments. Each sample harbored the C228T variant of the *TERT* promoter region as well as the fusion t(12;16) with its reciprocal breakpoints. (C–F) The results for the tumors from patients 2–5 are presented the same way. Most different mutations were detected in patient 4 with a very pronounced heterogeneous distribution pattern. MLS indicates myxoid liposarcoma.

In primary tumors, an MLS‐defining translocation was detected in each tumor (Figure 1A), with all samples of each tumor sharing the same breakpoints. However, 6.5% of samples (11 of 170) with low tumor content showed no tumor‐specific mutations (Figures 3A,B and 4B). For t(12;16), both breakpoints of the reciprocal translocation were present; whereas, for t(12;22), only one breakpoint was detected. This likely results from the fusion process forming a dicentric chromosome and an acentric chromosome, with the acentric chromosome lost during cell division (see Figure S2D).^19^ Each SV was validated with the design of a patient‐specific reference fusion sequence (see Materials and Methods, above).

In two tumors (patients 11 and 12), alternative fusions of *DDIT3* and *FUS* were detected. Patient 11 had an additional breakpoint in *ZNF213* on chromosome 16, leading to the translocation t(12;16;16)(q13;p11;p13.3) (see Figure S2B). Patient 12 harbored an additional breakpoint in *THBS1* on chromosome 15, resulting in t(12;15;16)(q13;q14;p11) (see Figure S2C).

**FIGURE 2:**
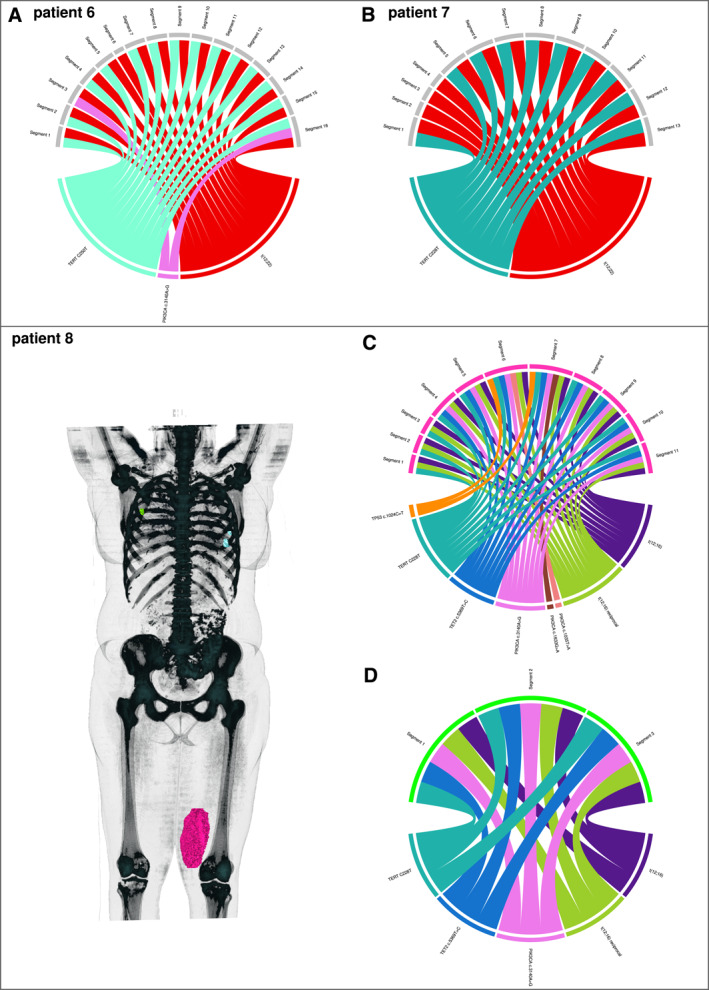
Tumor heterogeneity in MLS, part 2, and metastatic disease in patient 8. Each chord diagram has the same structure as described for Figure 1. (A,B) The MLS tumors that had fusion of *DDIT3* and *EWSR1* t(12;22) are depicted (patients 6 and 7). In every segment, the individual and fusion‐specific breakpoint was detected. The reciprocal breakpoint could not be detected in any of the tumors. Once again, the *TERT* promoter region was very dominant, with proven variants in almost all segments (C250T in patient 5 and C228T in patient 6). (C,D) These chord diagrams show two different tumors from the same patient. On the left, three‐dimensional reconstructions of each tumor were merged into a three‐dimensional reconstruction of a PET‐CT scan for better visualization. (C) Patient 8 initially presented with a high‐grade MLS in his left thigh (pink tumor). After resection, metastases in the lungs (blue tumors) and breast (green tumors) were observed during follow‐up care 6 months postoperatively and (D) were resected accordingly (green tumor). The primary tumor showed marked intratumoral heterogeneity regarding single‐nucleotide variants (*PIK3CA*, *TET2*, and *TP53*). The patient‐specific translocation t(12;16) and a variant in the *TERT* promoter region (C228T) were detected in every segment. (D) The metastasis exhibited the same mutation profile as some segments of the primary tumor. MLS indicates myxoid liposarcoma; PET‐CT, positron emission tomography‐computed tomography.

The variants C228T and C250T in the *TERT* promoter region were the next most common event (83.3% of patients), whereas C228T was observed in 67% of patients (12 of 20 tumors, 73 of 170 samples; Figure 1A). Only patients 2 and 5 had no detectable *TERT* promoter variant. One tumor harbored both variants, but they were mutually exclusive and occurred in different samples (Figure 1D). Otherwise, the distribution within a tumor was consistent, with only a few segments showing no mutation. Variants in *PIK3CA* were detected in 75% of patients (14 of 20 tumors, 66 of 170 samples). The most common variant was c.3140A>G, which was observed in 42% of patients (six of 20 tumors, 20 of 170 samples). SNVs in *PIK3CA* were more heterogeneous and occurred randomly distributed within each tumor, with some samples harboring multiple variants (Figure 2C). Otherwise, only a few additional SNVs were detected in *PTEN*, *PTENP1*, *TET2*, and *TP53* (Figures 1A,C,E and 2C).

In conclusion, each patient's translocation remained consistent across all samples, whereas SNVs, particularly those in *PIK3CA*, showed significant heterogeneity.

### Correlation between *TERT* promoter mutation status and clinicopathologic parameters

To place the results on the prevalence of mutations in the *TERT* promoter from the current study in the context of previous findings,^20^, ^21^ clinical and pathologic data from the patients and tumors were evaluated. For each patient, data on the presence of metastases, age at diagnosis, tumor size, tumor grading (according to the National Federation of Centers for the Fight Against Cancer), and therapy‐naive tumor necrosis was collected (Table 2). Mutations in the *TERT* promoter region (C228T or C250T) were found in 83.3% of patients (10 or 12). The prevalence of *TERT* hotspot mutations did not correlate significantly with any of the analyzed clinicopathologic parameters (Fisher exact tests).

**TABLE 2:** Correlation of the *TERT* promoter variant with clinicopathologic parameters in patients with myxoid liposarcoma.

*TERT* hotspot mutation: C228T or C250R	No. of patients	
	Mutation, *n* = 10	Wild type, *n* = 2
Metastases		
No metastasis (M0)	5	1
Metastasis (M1)	5	1
Tumor grade, FNCLCC		
1	3	0
2	6	2
3	1	0
Tumor size, cm		
<10	2	0
≥10	8	2
Therapy‐naive tumor necrosis, %		
No necrosis	3	1
<50%	6	0
≥50%	0	1
Age at diagnosis, years		
<50	4	1
≥50	6	1
Follow‐up		
Mean follow‐up, months	60	22

*Note*: Correlation of *TERT* promoter mutation status with clinicopathologic parameters in *n* = 12 patients who had MLS treated at the Freiburg Comprehensive Cancer Center. Statistical analysis with the Fisher exact test indicated no significant correlation of *TERT* promoter mutation status with any of the parameters. No data on tumor necrosis were available for one patient.

Abbreviations: MLS, myxoid liposarcoma; FNCLCC, Fédération Nationale des Centres de Lutte Contre le Cancer.

Analyzing the heterogeneity of metastases provides insight into clonal evolution

**FIGURE 3:**
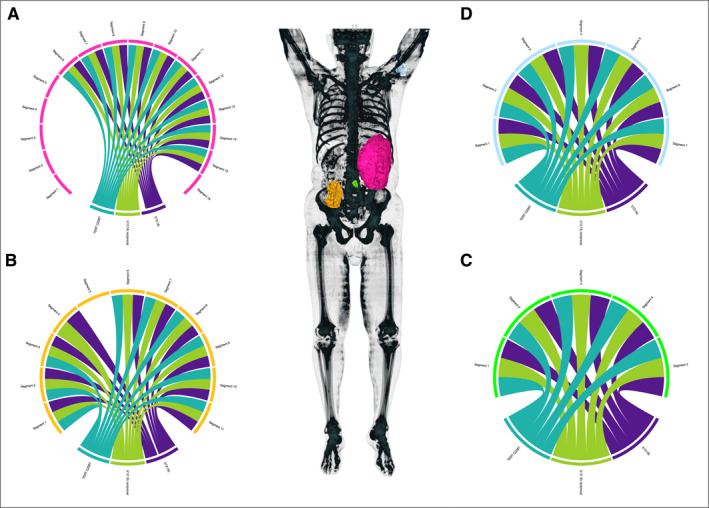
Tumor heterogeneity in metastatic disease (from patient 9 with MLS). The chord diagrams show different tumors from the same patient. The structure of the diagram is the same as described for Figure 1. In the center, three‐dimensional reconstructions of each tumor were merged into a three‐dimensional reconstruction of a PET‐CT scan for better visualization. Patient 9 initially presented with incomplete resection of a high‐grade MLS in the right thigh. After wide resection, a retroperitoneal metastasis (A) was observed 3 years postoperatively and was resected consecutively (pink tumor) followed by multiple recurrences and resections over a period of approximately 6 years, with (B) another retroperitoneal metastasis (orange tumor), (C) a mesenterial metastasis (green tumor), and (D) a metastasis in the right upper arm (light‐blue tumor). The same variants were detectable in each segment of a tumor that had positive detection of tumor mutations. This was consistent even for metastatic disease. The lack of detected tumor mutations in a few segments was probably caused by a lack of tumor cells in the tissue used for extraction rather than by genomic heterogeneity. MLS indicates myxoid liposarcoma; PET‐CT, positron emission tomography‐computed tomography.

**FIGURE 4:**
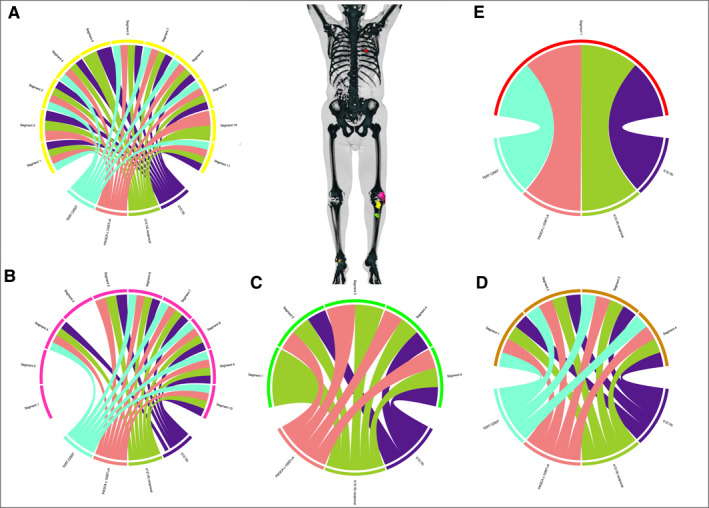
Tumor heterogeneity in metastatic disease (from patient 10 with MLS). The chord diagrams show different tumors from the same patient. The structure of the diagram is the same as described for Figure 1. In the center, three‐dimensional reconstructions of each tumor were merged into a three‐dimensional reconstruction of a PET‐CT scan for better visualization. Patient 10 presented after multiple marginal resections and recurrences of a high‐grade MLS in both lower legs. (A) With no sign of distant metastasis, another resection was performed (yellow tumor). (B–D) The resection was followed by multiple local recurrences and resections (pink, green, and brown tumors) until, finally, (E) a lung metastasis was detected and resected 2 years after first presentation (red tumor). In all samples, the same patient‐specific translocation as well as the *PIK3CA* variant c.1035T>A were detected with few exceptions. In contrast, the *TERT* promoter variant C250T was present in every tumor except tumor 3 (local recurrence; green tumor in C), showing correlation of the mutation profile with a few segments in tumors 1 and 2 (A,B). MLS indicates myxoid liposarcoma; PET‐CT, positron emission tomography‐computed tomography.

To determine the extent to which primary tumors and their respective metastases harbor the same cancer driver mutations, 84 samples from 11 tumors (primary lesions and metastases) in three patients were examined as described above (Figures 2, 3, and 4).

Patient 8 (Figure 2C) initially presented with high‐grade MLS (grade 2) in the left thigh (Figure 2C, pink tumor). The tumor was primarily resected (40% tumor cell necrosis), followed by adjuvant radiotherapy. Six months after primary resection, synchronous metastases in the lungs and the breast were resected, and the tissue of the breast metastasis was examined (Figure 2D, green tumor). No tissue was available for the analysis of the lung metastases (Figure 2D, light‐blue tumors). The primary tumor showed marked intratumoral heterogeneity with respect to SNVs (*PIK3CA*, *TET2* and *TP53*). The patient‐specific translocation t(12;16) and the *TERT* promoter variant C228T were detected in each sample. All three samples of the metastasis showed the identical translocation t(12;16) and the same variants in the *TERT* promoter region, *PIK3CA*, and *TET2* that were present in the primary tumor samples 4, 5, 8, 9, 10, and 11. This suggests that the metastasis originated from a subclone of one of these areas.

Patient 9 (Figure 3) initially presented with an incomplete resection of a high‐grade MLS in the right thigh. Subsequently, a complete resection was performed in our department. A retroperitoneal metastasis was identified 3 years after surgery (Figure 3A, pink tumor), followed by multiple recurrences and resections over a period of 6 years: Another retroperitoneal metastasis (Figure 3B, orange tumor), a mesenterial metastasis (Figure 3C, green), and a metastasis in the right upper arm (Figure 3D, light‐blue tumor) were identified. Histopathologic examination revealed a tumor cell necrosis ranging from 0% to 50%. Thirty‐nine samples from four metastases were analyzed. Except for the marginal segments that had little or no tumor content, the same variants were detected in each segment of the first metastasis (Figure 3A, pink tumor): the patient‐specific translocation t(12;16) and the *TERT* promoter variant C228T. This was consistent in the remaining metastases. The only exception were samples 4 and 5 in the second metastasis (Figure 3B, orange tumor). Because the mean coverage in the segments without tumor variants was not significantly different from the others, it is reasonable to assume that there were hardly any tumor cells in the extracted tissue from these segments. No other variants were detected in any of the samples.

Patient 10 (Figure 4) initially presented after multiple marginal resections and recurrences of a high‐grade MLS in both lower legs. With no evidence of distant metastases, resection of a local recurrence in the left leg was performed (Figure 4A, yellow tumor). Then, multiple local recurrences and soft tissue metastases in the other leg were completely resected (Figure 4B–D, pink, green, and brown tumors). Two years after initial presentation, a lung metastasis was detected and resected (Figure 4E, red tumor). Thirty‐one samples from five recurrences/metastases were analyzed. In all of these tumors, the same patient‐specific translocation was detected (Figure 4A–E). In addition, the *PIK3CA* variant c.1035T>A was present in every tumor and every sample with detectable translocation. In contrast, the *TERT* promoter variant C228T was not present in tumor 3 but was present in every other tumor (Figure 4C, green tumor). The coverage of the *TERT* promoter region did not differ significantly between the tumors (ordinary one‐way analysis of variance; see Figure S1C). In terms of temporal‐spatial tumor evolution, these results suggest that tumor 3 developed from a different subclone than the other three tumors. Therefore, different clones had acquired the ability to detach from the primary tumor and form metastases.

These findings further confirm that the translocation is the primary tumor driver and remains stable throughout tumor evolution.

## DISCUSSION

STS are highly heterogeneous at the macroscopic, cellular, and therapeutic response levels.^22^ Tumor regions may behave differently based on their (epi)genetic signature. Metastases inherit this signature from their primary tumor clone.^23^ Previously, we demonstrated in two patients with MLS that genetic heterogeneity was present in primary tumors.^6^ To further understand MLS development and therapeutic options, a cohort of 170 samples from 20 tumors across 12 patients with MLS were analyzed for genetic heterogeneity with respect to driver mutations.

DNA from samples with histopathologically proven MLS were enriched with a subtype‐specific target‐enrichment panel (*MLS target enrichment panel*).^6^ This assay covers only a small fraction of the genome that included all regions with a mutation frequency >5% in previous studies^24^ as well as the intronic sequences of the MLS‐specific breakpoints. Any conclusions, therefore, are limited to these regions. Whole‐genome sequencing of each sample would be required to fully examine intratumoral heterogeneity. However, because MLS harbors only few additional mutations beyond typical translocations, this targeted approach is a reasonable compromise.^20^, ^25^, ^26^, ^27^, ^28^

In the analysis of SVs in MLS tumors, the common translocations t(12;16) and t(12;22) were consistently observed, with t(12;22) slightly more prevalent than previously reported.^6^, ^29^ For t(12;22) events, only one breakpoint of the reciprocal translocation was identified in all samples. After examining the fusion sequences, this is likely caused by the formation of a dicentric fusion chromosome and an acentric fusion chromosome, with the latter lost during cell division.^19^ Notably, two tumors with t(12;16) (patients 11 and 12) had a rare translocation involving ZNF213 on chromosome 16 and THBS1 on chromosome 15. Although variants were cross‐checked using patient‐specific reference fusion sequences, validation relied on the same data set.

The presence of a single t(12;16) or t(12;22) in all tumors from a patient supports the hypothesis that primary, multifocal MLS is not caused by multiple primary tumors but represents a metastatic disease that is only diagnosed at this late tumor stage.^30^ Every histopathologically confirmed MLS sample that had sufficient tumor content contained one of these translocations. Nevertheless, there were some segments without detectable tumor mutations (e.g. Figures 3 and 4). This was likely caused by minimal tumor cells rather than an absence of the translocation because variant detection in other segments was consistent even with low coverage. Thus the results of these mutation‐negative samples did not contradict our hypothesis that the translocations are present in every tumor cell. The persistence of tumor‐specific SVs in all segments and metastases underscores their role in tumor initiation and progression. These findings support translocation as the key event in tumorigenesis, followed by mutations in the *TERT* promoter and other cancer drivers.

Variants in the *TERT* promoter occurred in 85% of tumors and remained consistent within each. Except for one tumor that had both C228T and C250T (Figure 1E), these variants were mutually exclusive. The prevalence of *TERT* hotspot mutations was consistent with previous reports.^20^, ^21^ Unlike other malignancies, the occurrence of these mutations in MLS does not significantly correlate with prognostic clinicopathologic parameters or metastatic potential. This was demonstrated in previous studies and in our data set, although the statistical power of the latter is very limited because of the small sample size. This underscores the critical role of telomerase reactivation as an early event in the tumorigenesis and development of MLS, secondary to the initiating translocation event.^31^ However, our analysis of intratumoral heterogeneity now reveals that mutations in the *TERT* promoter not only occur with a high prevalence but are distributed homogeneously within individual tumors. Therefore, if a mutation in the *TERT* promoter is detected in an MLS, it can be concluded from the available data that this mutation is probably distributed throughout the entire tumor. This suggests that *TERT* promoter mutations could serve as a promising therapeutic target in MLS treatment.

MLS has been linked to activating *PIK3CA* mutations and PI3K/Akt pathway alterations, with previous studies reporting a 15%–20% mutation rate.^32^ At first glance, this seems to contrast with our prevalence of 75%. However, our data indicate that different *PIK3CA* mutations with a very heterogeneous mutational landscape appear in MLS. Thus the high prevalence of *PIK3CA* mutations may have been underestimated in previous studies because of limited hotspot analysis and single‐sample testing per tumor.

Our findings have important implications for MLS treatment. Although localized tumors respond well to multimodal therapy, metastatic disease is often devastating because of the poor response to traditional systemic therapies.^33^ Targeted therapies for recurrent mutations are eagerly awaited. Research has focused on PIK3CA inhibitors, especially because some are already approved by the US Food and Drug Administration. A trial with copanlisib has produced a partial response in a patient who had an MLS.^34^ However, our data indicate that PIK3CA is not an ideal target because activating *PIK3CA* mutations are often not present throughout the primary tumor or its metastases. Thus, at best, a partial response can be expected, with most tumor cells escaping treatment. In contrast, *TERT* promoter mutations may be a more favorable target because they appear more consistent throughout tumor progression. Although direct TERT inhibitors are unavailable, alternative strategies have been successfully developed to target *TERT* promoter–mutated tumor cells through the regulation of *survivin* and *TRAIL‐R2.* ^35^

Therapeutic targeting of the t(12;16) or t(12;22) fusion protein, like t(9;22) in chronic myeloid leukemia, would be ideal because tumor cells depend on its presence for tumor growth. However, the FUS::DDIT3 protein is located in the cell nucleus and thus is not accessible to neutralizing antibodies.^36^ Nevertheless, a promising approach for fusion‐driven tumors in the era of gene therapy is the site‐specific integration of a suicide gene by Cas9.^37^

## CONCLUSION

In conclusion, our data provide insight into the intratumoral heterogeneity of MLS and their clonal evolution over time, which is highly relevant for the selection of targets and the development of therapeutic approaches and thus can serve as basis for further research.

## AUTHOR CONTRIBUTIONS

**Adrian Schmid**: Conceptualization, writing–review and editing, writing–original draft, project administration, visualization, formal analysis, data curation, software, methodology, and investigation. **Anja E. Eisenhardt**: Conceptualization, data curation, formal analysis, and writing–review and editing. **Balazs Bogner**: Data curation, writing–review and editing, and visualization. **Alexander Runkel**: Data curation, writing–review and editing, and investigation. **Ute Lausch**: Methodology, data curation, investigation, and writing–review and editing. **Thomas Pauli**: Data curation, methodology, and writing–review and editing. **Laura N. Antolini**: Data curation and writing–review and editing. **Anika Boneberg**: Data curation and writing–review and editing. **Jurij Kiefer**: Data curation, investigation, and writing–review and editing. **Peter Bronsert**: Data curation and writing–review and editing. **Melanie Boerries**: Data curation and writing–review and editing. **Steffen U. Eisenhardt**: Data curation, funding acquisition, and writing–review and editing. **David Braig**: Conceptualization, methodology, data curation, investigation, supervision, project administration, writing–review and editing, funding acquisition, formal analysis, and validation.

## CONFLICT OF INTEREST STATEMENT

The authors disclosed no conflicts of interest.

## Supporting information

Supplementary Material

Figure S1

Figure S2

## Data Availability

The data sets used and/or analyzed during the current study are available from the corresponding author on reasonable request.
