# Supplementary material for: Intratumoral heterogeneity of cancer driver genomic alterations in myxoid liposarcomas
Source: Cancer. 2025 Jun 9;131(12):e35937. doi: 10.1002/cncr.35937 (PMC12148203; doi:10.1002/cncr.35937)
Supplement: Supplementary file 1 — Supplementary Material [file CNCR-131-e35937-s003.docx]

**FIGURE S1** Sequencing statistics and metrics for MLS tumor samples. The statistics were calculated for MLS tumors after enrichment with the subtype specific MLS panel. **(a)**On-target rates after target enrichment were calculated for each segment of each tumor. With few exceptions, the on-target rates after double hybridization capture were > 90 %. **(b)** Shows the comparison of mean on-target rates for each tumor and the corresponding matched normal sample for patients, where the required samples were sequenced in the same sequencing run on the same sequencer. There was no significant difference between tumor and matched normal samples (Wilcoxon matched-pairs signed rank test). **(c,d)** The same statistics were calculated for target region mean coverages. Again, every segment of each tumor was analyzed separately. In terms of coverage, there were significant differences between and within patients (ordinary one-way ANOVA, Tukey’s multiple comparisons test; paired t-test). This time, a significantly higher coverage was observed for matched normal samples sequenced in the same sequencing run and the same sequencing machine (paired t-test). **(e)** Target region mean coverage was significantly lower in *TERT* promoter region, aggravating variant detection in this region (one-way ANOVA, Tukey’s multiple comparisons test). **(f)** This drop in coverage in the *TERT* promoter region is most likely due to high GC-content in this area. In this sense, there was significant correlation between coverage and GC content in some samples (Pearson test).

**FIGURE S2** Schematic visualization of detected fusion events in MLS. **(a–d)** This shows a schematic visualization of the detected fusion events in MLS tumors. **(a)** Shows the common fusion event of a t(12;16) reciprocal translocation, as it occurs in most MLS. Two aberrant fusions were detected in two patients. Patient 11 **(c)** showed an additional fusion of FUS to ZNF213 on chromosome 16. In the MLS of patient 12 **(d)**, an additional fusion to THBS1 on chromosome 15 was detected. **(d)** The fusion sequences of t(12;22) events suggest formation of an acentric and a dicentric chromosome, with loss of the acentric chromosome in cell division.
