# Supplementary figures and images for: Intratumoral heterogeneity of cancer driver genomic alterations in myxoid liposarcomas
Source: Cancer. 2025 Jun 9;131(12):e35937. doi: 10.1002/cncr.35937 (PMC12148203; doi:10.1002/cncr.35937)

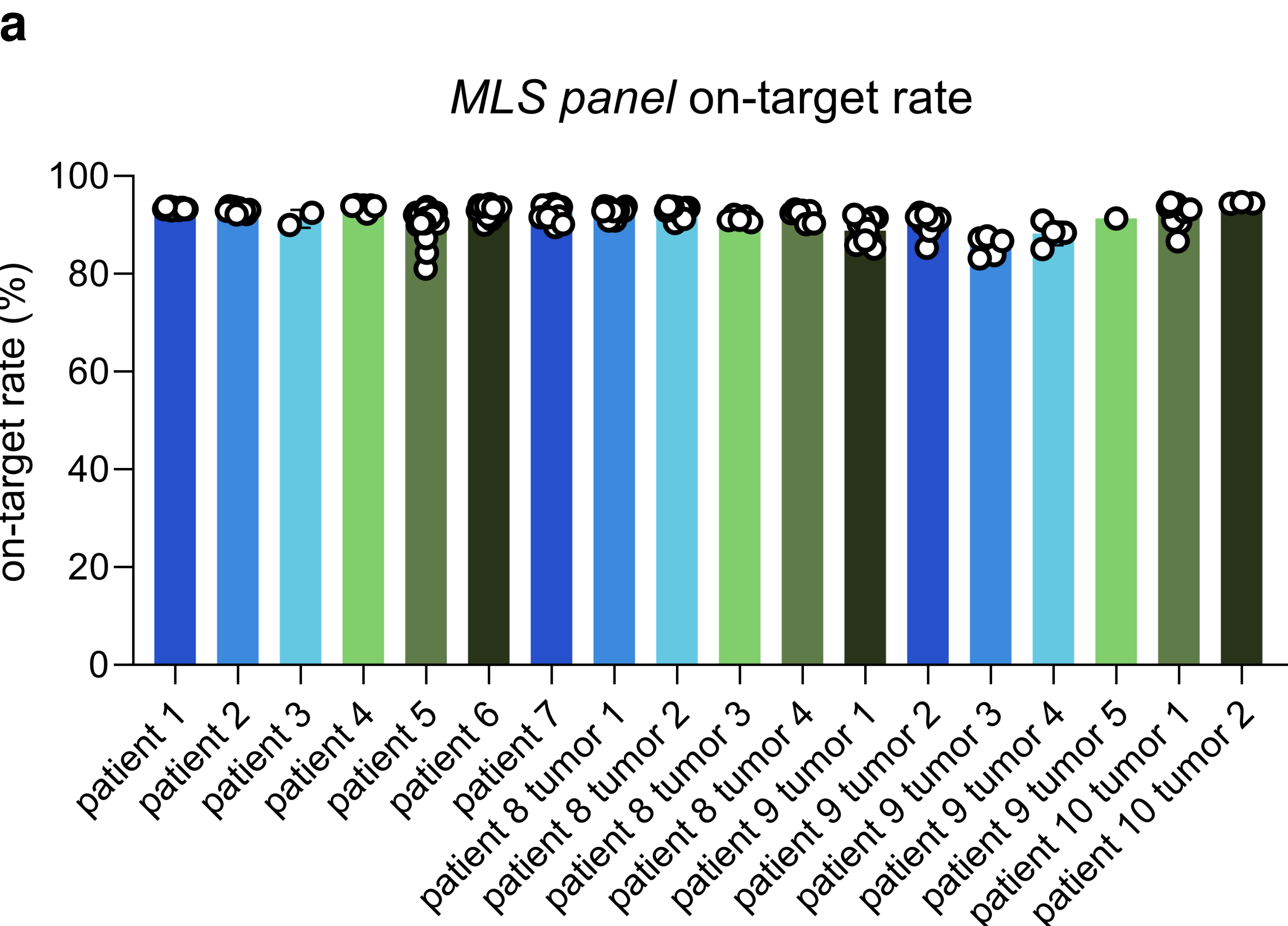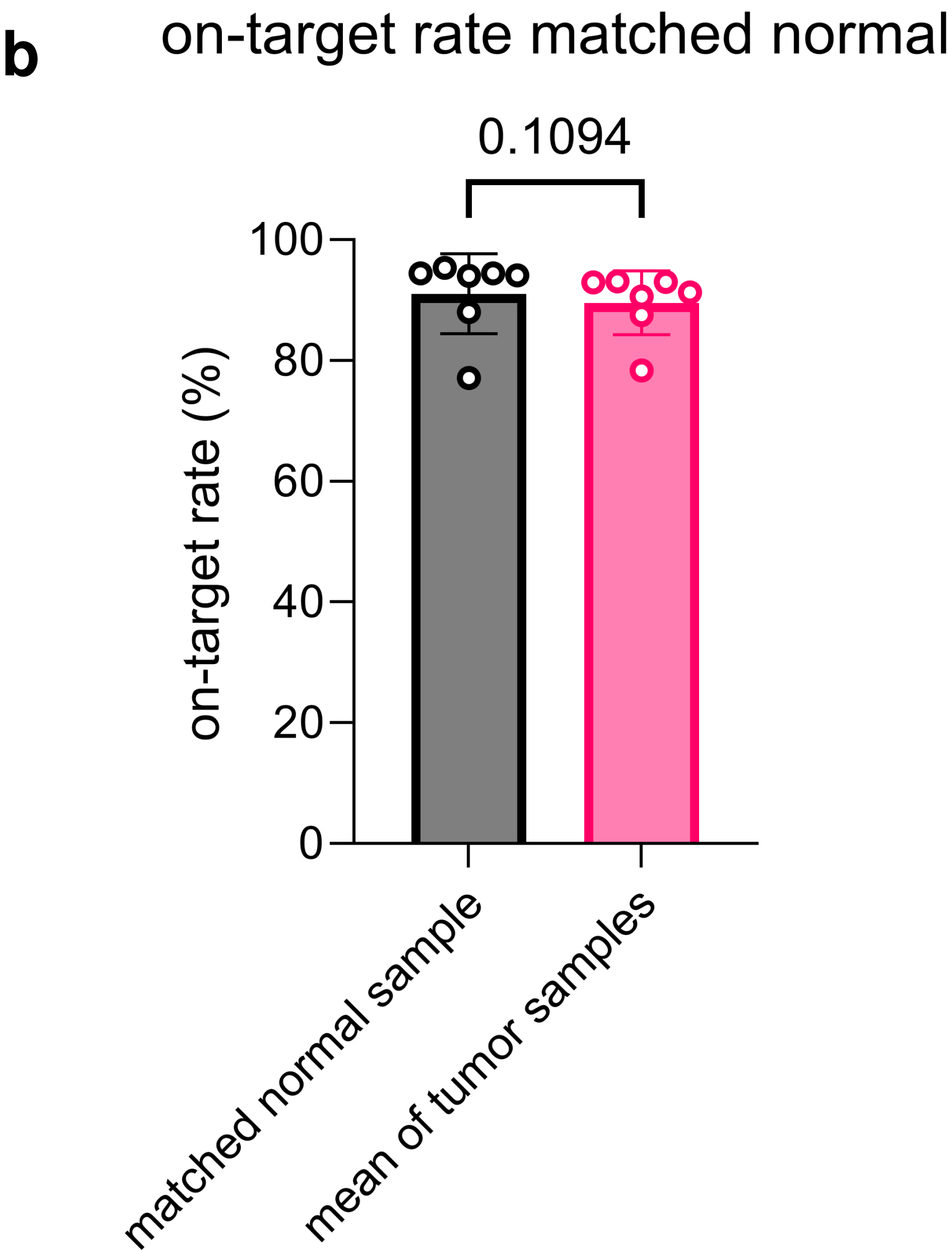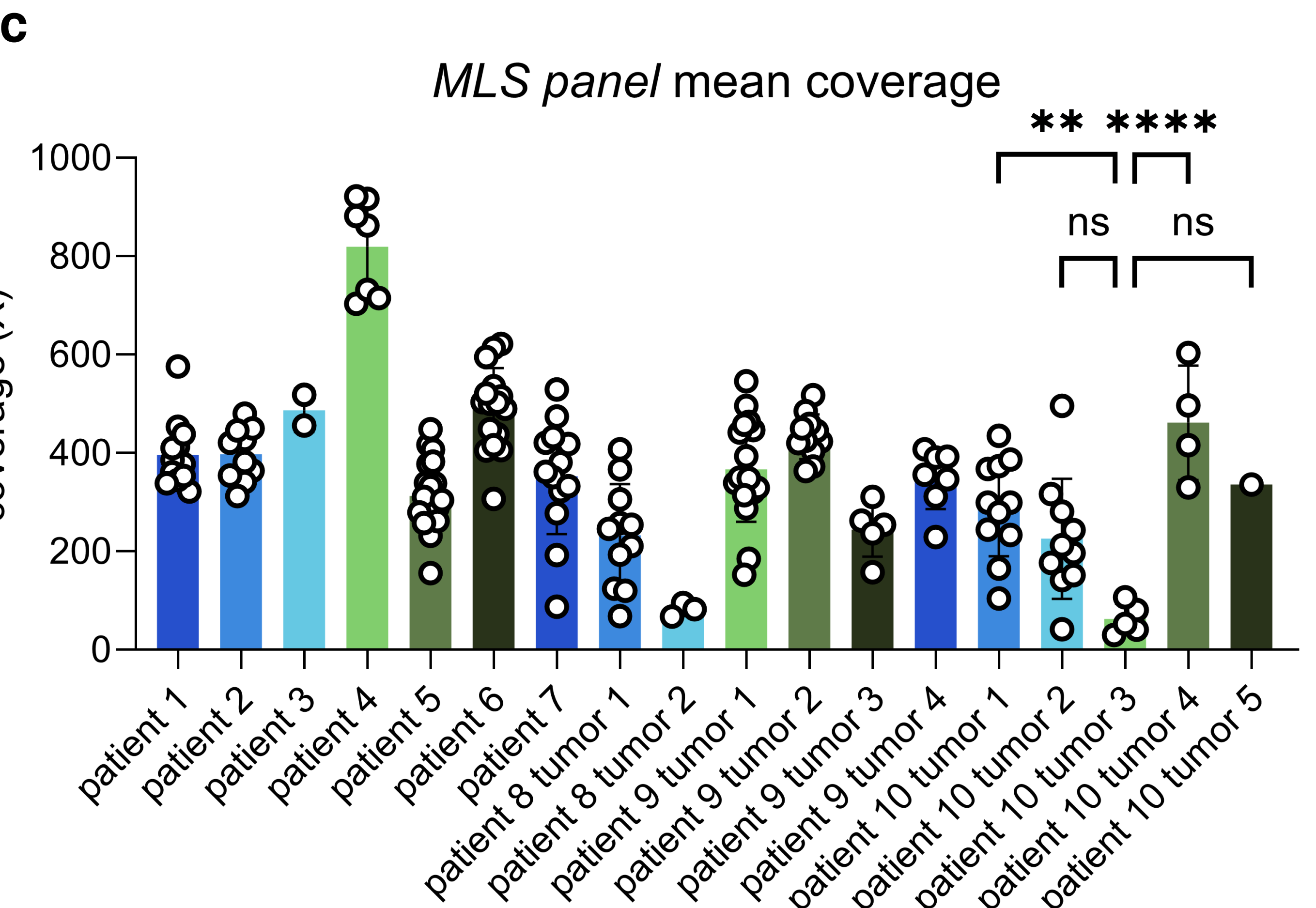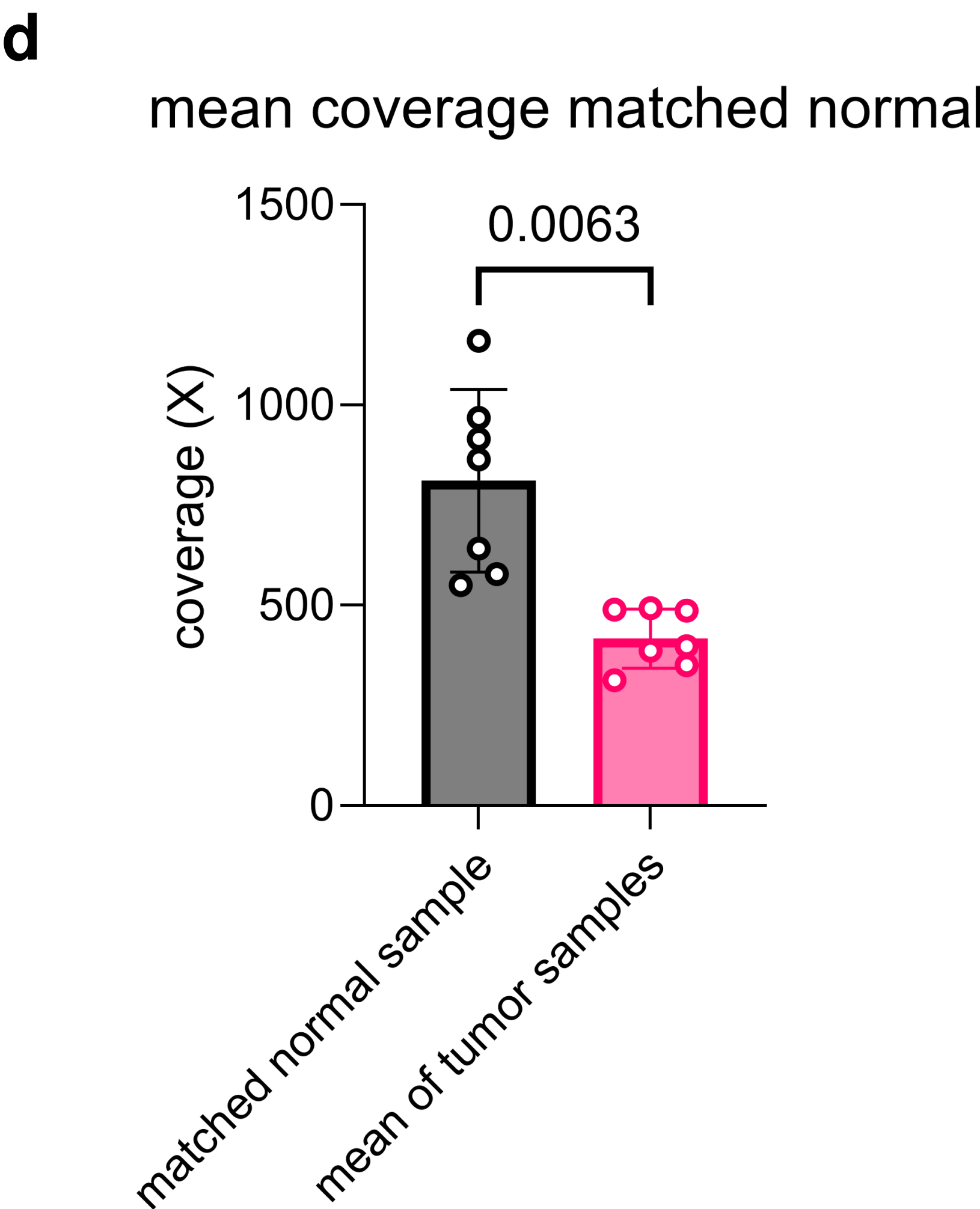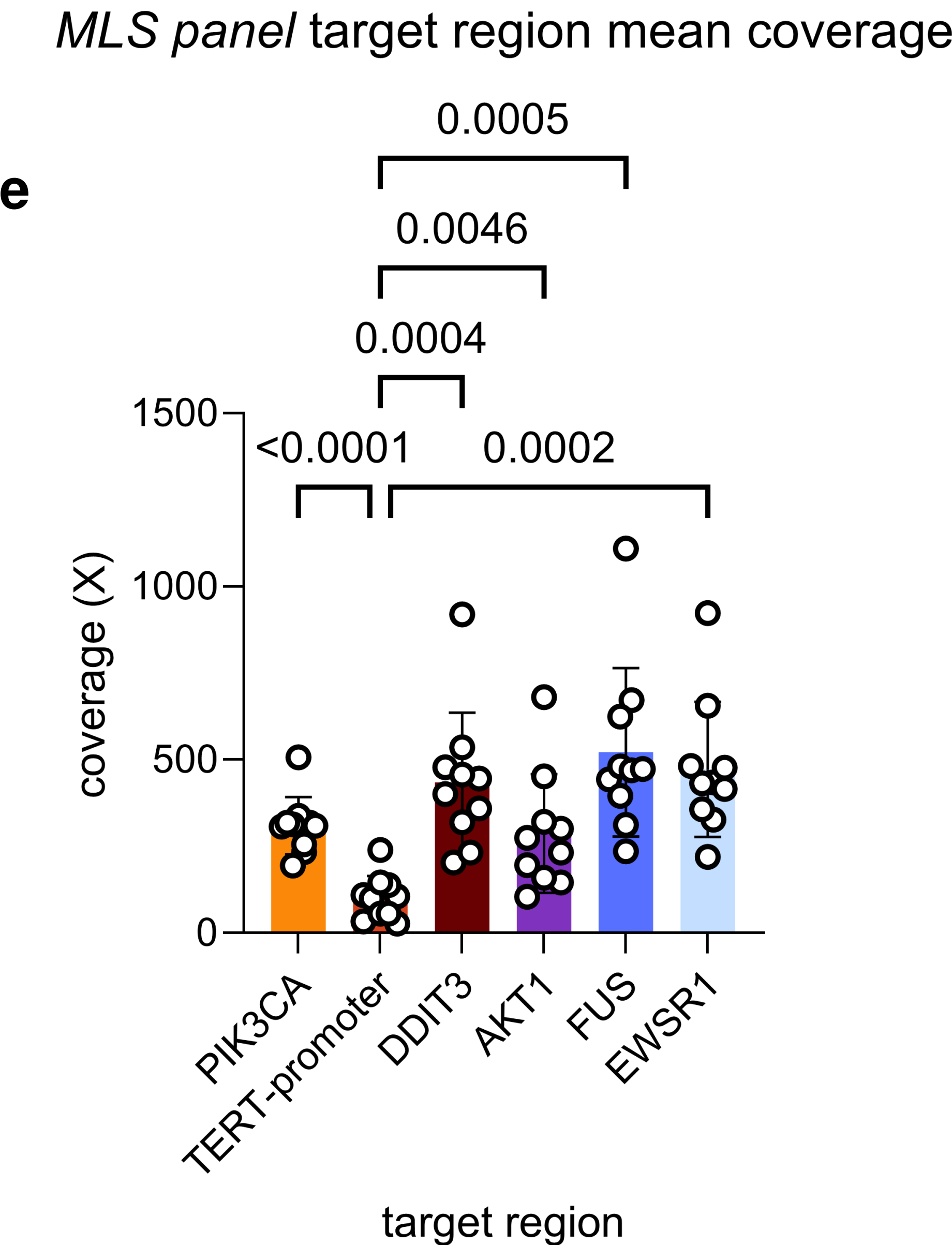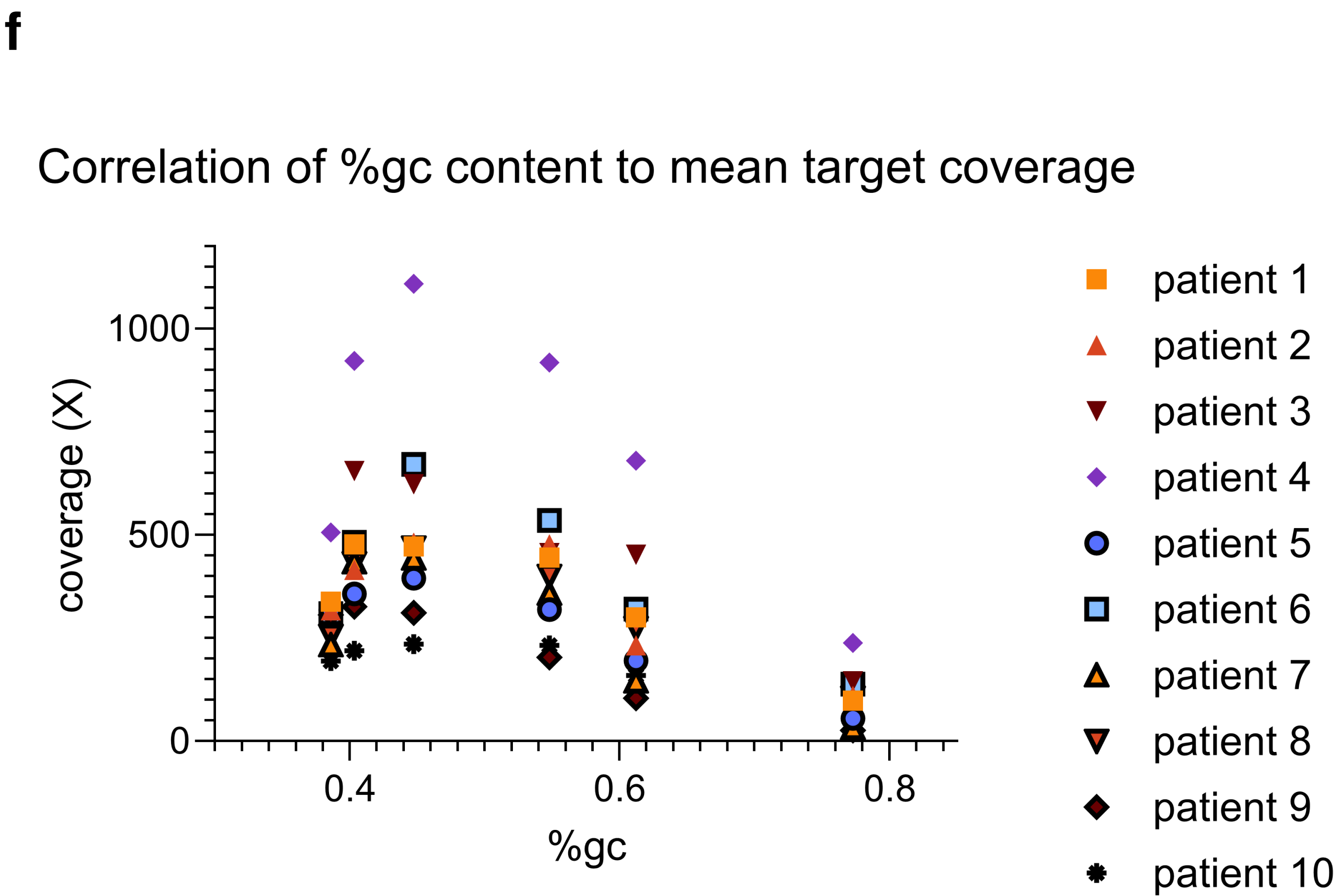

Supplement: Supplementary file 2 — Figure S1 [file CNCR-131-e35937-s001.pdf]

Supplementary figure 2

a

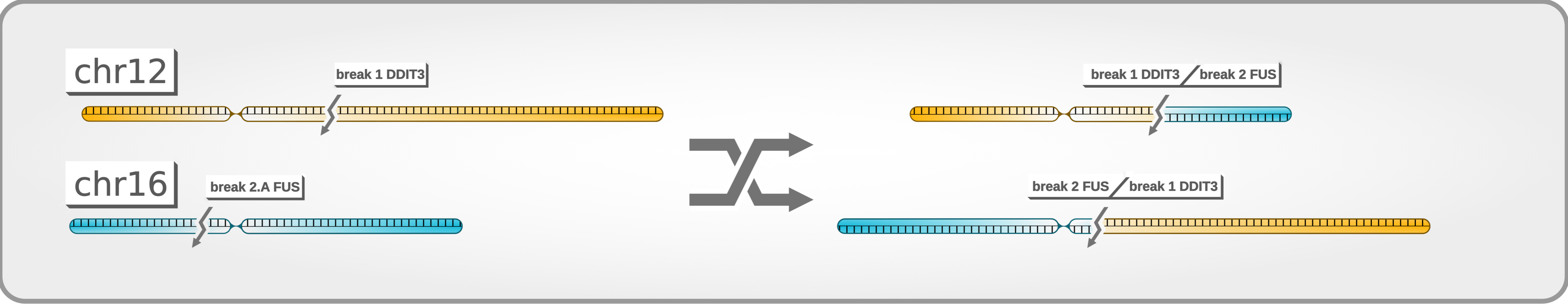

b

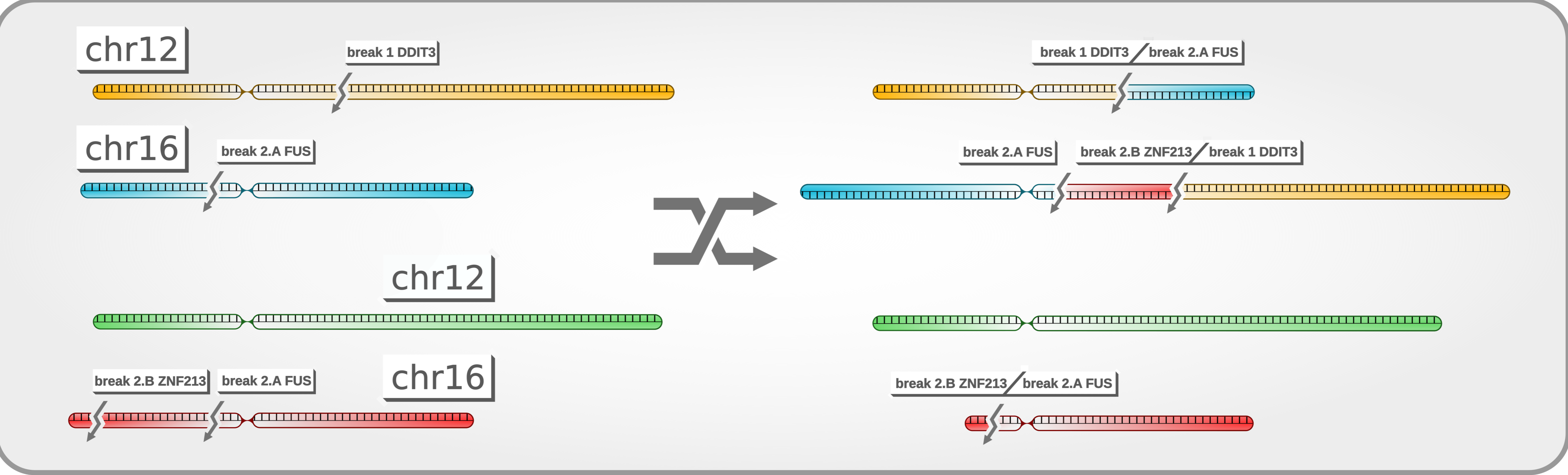

c

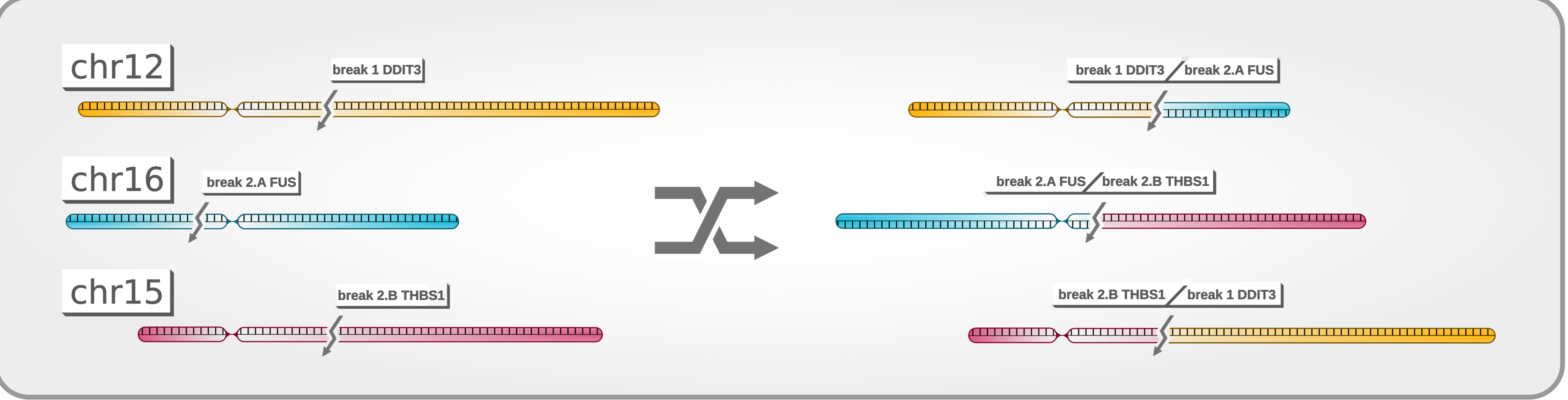

d

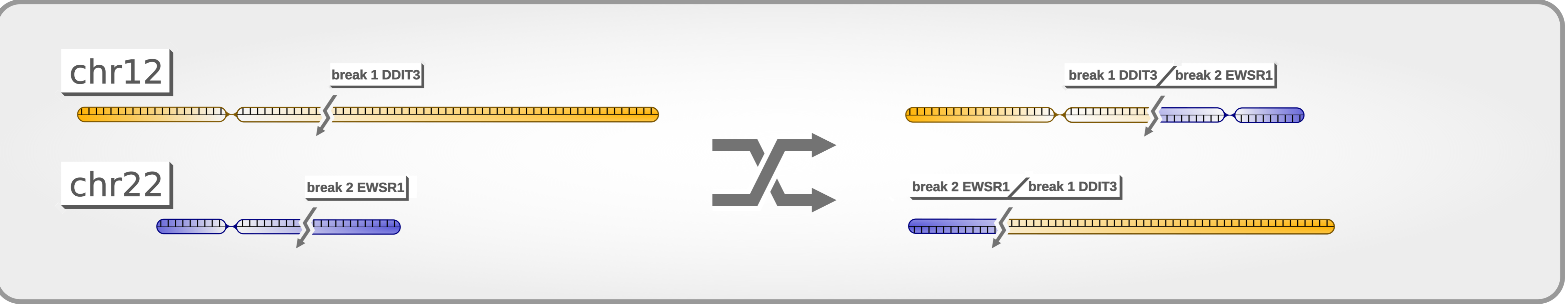

Supplement: Supplementary file 3 — Figure S2 [file CNCR-131-e35937-s002.pdf]
